# The Importance of Different Species of *Aspergillus* for Stimulation of Plant Growth

**DOI:** 10.3390/plants15192943

**Published:** 2026-09-26

**Authors:** Mohamad Hesam Shahrajabian, Mengyuan Zhang, Jiayi You, Yuheng Li, Haopeng Yang, Jianxiang Wang, Na Wang, Wenli Sun

**Affiliations:** National Key Laboratory of Agricultural Microbiology, Biotechnology Research Institute, Chinese Academy of Agricultural Sciences, Beijing 100086, China; hesamshahrajabian@gmail.com (M.H.S.); 18234025093@163.com (M.Z.); 15140339727@163.com (J.Y.); 19260389182@163.com (Y.L.); 18641257508@163.com (H.Y.); 18741251078@163.com (J.W.); 15850660098@163.com (N.W.)

**Keywords:** *Aspergillus* spp., biostimulant, fungal endophytes, plant growth-promoting fungi, sustainability

## Abstract

Recently, the application of filamentous fungi which can improve the growth of different plants has been considered as an important alternative to the application of synthetic fertilizer in modern sustainable agricultural practices. Plant growth-promoting fungi (PGPF) are fungus residents in the rhizosphere which are beneficial to plants, and the association between these fungi and plants can improve plant growth. Species of *Aspergillus* have been used in the promotion of plant growth of many agricultural and horticultural plants. The plant growth promotion activity of *Aspergillus* species can induce better absorption of mineral nutrients through deep root penetration, maintenance of soil pH, and production of siderophores; improved physicochemical processes through delayed senescence, increased photosynthesis, improved stomatal conductance, and improved water-holding capacity; enhanced morphological growth via regulation of phytohormone levels; better reproductive potential and yield through improved physiological and physical growth; higher abiotic stress tolerance through upregulation of stress-ameliorative genes; and better induction of disease resistance. Sustainability is the main driving force for the application and the investigation of biocontrol with plant growth-promoting fungi. The present review article aims to present the most up-to-date results and findings regarding the practical applications of *Aspergillus* spp. in agricultural and horticultural species. Moreover, the mechanisms of action of these species of fungi are briefly described.

## 1. Introduction

Plant biostimulants are particular products used to promote crop production, and are quickly becoming common in both the chemical marketplace and the agricultural seed industry [1,2,3,4,5], and they are described as plant biostimulants, plant probiotics, biofertilizers, metabolic enhancers, and biostimulators [6,7,8]. Plant growth-promoting microorganisms (PGPMs) include fungi, bacteria, and other microorganisms, which improve the performance of plants as well as increase nutrient absorption under both abiotic and biotic stress conditions, with notable potential advantages for the growth and development of seedlings [9,10]. Plant growth-promoting fungi (PGPF) are a group of non-pathogenic soil-borne filamentous fungi which have beneficial impacts on plants, as they can enhance plant growth both indirectly and directly by producing phytohormones, fixing nitrogen, and inducing systemic resistance [11,12,13]. Plants are always prone to interacting with different microbes in several ways, which include symbiotic and phytopathogenic associations, and the interactions may significantly influence agriculture, the environment, and ultimately, the economy. Interactions between fungi and plants can be highly effective in shaping plant community composition and diversity due to fungi’s influence on plants directly, as well as indirectly by influencing facilitation and competition [6,7,8]. The outcome of plant–fungi interactions can be affected by different environmental factors, such as light, temperature, CO_2_, water, pollutants, and nutrient concentration in the soil, which can modulate the balance between antagonism and mutualism [9,10].

*Aspergillus* is one of the most effectual organisms among numerous phosphate-solubilizing fungi which can dissolve insoluble phosphate and improve its uptake, which are the main causes of appropriate growth and development [14,15,16,17,18,19,20]. In many studies, it has been reported that a combination of biologically active compounds of plant or microbial origin with fungicides as well as their synthesized analogues is a promising and sustainable approach to control crop diseases [21,22,23,24,25]. *Aspergillus* species secrete various secondary metabolites known as mycotoxins into the environment, and mycotoxins are produced during consecutive enzyme reactions via different biochemically simple intermediary products from the primary metabolism of acetates, malonites, mevalonates, and some amino acids. *Aspergillus* species possess versatile characteristics enabling them to survive in different environmental conditions; the species is a ubiquitous fungal pathogen in a wide range of hosts, including animals and humans [21,22,23]. Aspergillosis is an infection caused by the mold *Aspergillus*; the illnesses resulting from aspergillosis infection usually influence the respiratory system, and allergic aspergillosis usually affects people with cystic fibrosis, bronchiectasis, and asthma; moreover, invasive aspergillosis usually affects people with weakened immune systems [6,7,8,9,10].

It is reported that the application of *Aspergillus* is an important strategy used by researchers to increase plant growth through stimulating iron transport to increase vegetative growth, stimulating substances responsible for pathogen suppression, inducing plant immunity, producing amino cephalosporanic acid acylase enzymes, controlling fungal phytopathogens, producing phytohormones, and stimulating plant growth by enhancing nutrient acquisition [14,15,16,17,18,19,20,21].

Fungi show significant metabolic properties because of their sophisticated genomic network, and they have notable importance for their roles in our world in different fields, such as their applications in agriculture, industry, and medicine. The filamentous fungi of the genus *Aspergillus* have been widely studied because they can interact with and colonize plant roots via different processes, which may increase plant growth through phytohormone synthesis, nutrient absorption, tolerance to abiotic stress, and induction of systemic resistance, as well as acting as biological control factors, which shows their value in protection of plants against different pathogens by inhibiting the growth of phytopathogens via the production of hydrolytic enzymes and antibiotics, and competing for space and nutrients. Their applications and their wonderful roles in environmentally friendly agricultural practices for different crops, such as lettuce, soybean, wheat, corn, tomato, beans, etc., have been mentioned in previous studies [26,27,28]. Species of the genus *Aspergillus* applied in the promotion of plant growth are *Aspergillus* spp. for cucumber, *A. terreus* for tomato and sunflower, *A. ustus* for *Arabidopsis thaliana* and tomato, *A. niger* for bean, coffee, lettuce, kale, melon, watermelon, scarlet eggplant, pepper and tomato, and *A. japonicus* for soybean and sunflower [29,30,31,32,33,34,35,36,37,38]. *A. terreus*, *A. fumigatus*, *A. niger*, *A. flavus*, and *A. oryzae* are among the most studied Aspergilli because of their agricultural, medical, and industrial importance [35,36,37,38]. The aim of this literature review is to survey the impacts of *Aspergillus* spp. on various agricultural and horticultural plants by considering successful paradigms and presenting cases studies. Furthermore, the major mechanisms of action of various plant growth-promoting fungi are briefly illustrated, along with a special section suggesting future needs which should be addressed for successful integration of plant growth-promoting fungi in horticultural and agricultural crop production. The practice of using competitive nontoxic *A. flavus* strains and *A. parasiticus* has led to the largest accomplishments in biocontrol of aflatoxin contamination discovered in both preharvest and postharvest produce to date. The adoption of nontoxic *Aspergillus* strains has usually led to massive diminutions in aflatoxin contamination in different field trials. Some of the most important virulence genes involved in the plant infection process in phytopathogenic fungi are cAMP-PKA pathways, cell wall synthesis- and degradation-related genes, factors associated with induction of plant defense tolerance and degradability, and autophagy pathways [26,27,28,29,30,31,32,33,34,35].

The information provided in this manuscript was obtained from randomized control experiments, review articles, and analytical studies and observations which were gathered from numerous literature sources such as Scopus, Google Scholar, PubMed, and Science Direct. The keywords used were the common and Latin names of various agricultural and horticultural species, fungal endophytes, plant growth-promoting fungi, *Aspergillus*, and microbial biostimulants, as well as biotic and abiotic stresses. This research examines the scientific literature from 1991 to September 2026 by conducting an analysis of the published sources in the literature. The screening process was done in three stages, namely screening of title, abstract and full text. The final search was completed on 10 September 2026, and 850 records from PubMed and Scopus were selected. Peer-reviewed original research articles and review papers directly relevant to the topics covered in this review were prioritized. Publications were selected according to their relevance to the topic. Duplicate studies and those that did not fall within the scope of the search were also excluded, and ultimately, 350 studies fulfilled the inclusion criteria. The beneficial effects of *Aspergillus* spp. in agricultural and horticultural industries are shown in Figure 1.

## 2. Fungal Endophytes

In recent years, fungal endophytes have been recognized as potential biostimulants which could improve commercial bio-prospecting of high-value secondary components of various plants [38,39,40]. They potentially spend their entire life in host plant tissues, either intra- or intercellularly, without any symptoms of infection in the host [41,42,43,44]. Endophytes are known as microorganisms which can be found inside the plant tissue without triggering any adverse impacts, resulting in a positive influence on crop yield and plant growth, as well as showing a high capacity to decrease the need for fertilizer and stimulate plant biochemical components by providing osmoregulation and antioxidant defenses, and affecting nutrient uptake effectiveness during several biotic and abiotic stresses [45,46,47,48,49]. Endophytes can increase photosynthesis, resulting in higher carbohydrate production and enhanced plant growth [45,46], and endophytic colonization can also change root morphology, increasing root branching and fine root hair growth [45,46,47]. Different endophytic bacteria and fungi, including *Bacillus*, *Actinomycetes*, *Trichoderma*, *Pseudomonas*, and *Epicoccum,* were reported to elicit plant disease tolerance in cacao, potato, chili, cotton, and tomato [50,51,52]. Endophytes produce siderophores, which have important functions in nitrogen fixation, scavenge ferric iron, and solubilize inorganic phosphate [53,54]. Siderophores can also support plant life in adverse stressful conditions [53,54,55]. It is reported that siderophores have significant applications in metal binding together with iron, and are ranked as Fe biosensors [53,54]. Siderophores are low-molecular-weight secondary metabolites produced by endophytes which have a high affinity for inorganic iron and function as metal chelators [45,46]. Microorganisms use siderophores to obtain the iron used in their cells, and so, during the breakage of its bond, they can release the P bound in the metal, making it assimilable for crops [53,54]. It is reported that endophytes produce siderophores which bind to insoluble (Fe ^3+^) ions, allowing the plant to absorb iron which would otherwise remain inaccessible [53,54,55]. There is increasing evidence for the activation of siderophore synthesis in bacteria in the presence of toxic metals, which suggests that siderophores can form stable complexes with other metals, such as Cd, Al, Cy, Pb, and Zn [52,53,54,55]. Increased siderophore secretion can be illustrated in two ways, by the direct involvement of heavy metals in the siderophore biosynthesis pathways and by heavy metals binding free siderophores and preventing iron chelation, resulting in Fe deficiency and therefore stimulating siderophore synthesis [50,51,52,53,54,55]. Moreover, along with hydroxamate- and carboxylate-type siderophores, bacteria may also produce catecholate- and mixed-type siderophores [53,54]. It is also very important to choose suitable fungal strains, as some strains are pathogens of crops and animals [55,56,57,58]: *A. tubingensis* is a pathogen of cotton [59], *A. niger* and *A. flavus* are phytopathogens of jojoba [60], and *A. aculeatus* may cause rot in grapes [61].

In recent years, the positive effects of inoculation with filamentous fungi in forage pastures have been reported, which can promote plant tolerance to salinity stress, enhance the quality of pastures, and improve the growth of pastures, with a positive influence on animal nutrition [62,63,64]. Plant–fungal interactions can be categorized as mutualistic, commensalistic, and pathogenic [65,66,67]. Fungal pathogens can change the plant’s capability to reproduce, survive, grow, compete, or defend itself against different parasites such as herbivores [68]; fungal–plant commensalism relates to the undisturbed existence of fungi within plant tissue and the interaction between the two species in which one experiences no net effect and one benefits [69,70,71]; and plants establish mutualistic interactions, usually known as symbiotic, with both eukaryotic and prokaryotic organisms [72,73,74]. The most important mechanisms of PGPF are the production of plant growth regulators such as abscisic acid, ethylene, gibberellins, cytokinins, and auxins, the production of organic acids, and mineral nutrients such as iron, zinc, potassium, phosphorus, and nitrogen, increased water uptake, the production of hydrolytic enzymes such as cellulases, pectinases, laccases, and xylanases, and reductions in the amount of ethylene, relief of various abiotic stresses in harsh environments, and induction of plant defense mechanisms against pathogens [75,76,77,78].

## 3. *Aspergillus* spp. as Plant Growth-Promoting Fungi

The genus *Aspergillus* contains different species such as *A. fumigatus*, *A. niger*, *A. flavus*, *A. oryzae*, *A. terreus*, * A. nidulans*, *A. brasiliensis*, *A. parasiticus*, *A. versicolor*, *A. sojae*, *A. ochraceus*, *A. sydowii*, *A. clavatus*, *A. glaucus*, *A. aculeatus*, *A. penicillioides*, *A. restrictus*, *A. amstelodami*, and *A. fischerianus* [79,80,81,82]. *A. glaucus* is a xerophilic fungus capable of growing at different temperatures and which can also grow as an outdoor fungus in winter and even in dry areas with low moisture content. *A. glaucus* can produce mycotoxin, which may cause pneumonitis and also dermatitis. *A. nidulans* is called a homothallic fungus because it can fertilize itself even without a mating partner, which has industrial importance as it produces enzymes like proteases, lipases, laccases, hemicellulases, and cellulases. Its colonial heads are short, columnar, and biseriate, and its conidiophores are usually short, brownish, and smooth-walled. Some of them serve as a plant growth stimulant via phosphate solubilization, nitrogen fixation, hydrogen cyanide production, and indole acetic acid production [83,84,85,86]. Mondal et al. [87] reported that a recently discovered soil fungus, *A. fumigatus* SKF-2, can produce a cocktail of carbohydrate debranching enzymes under solid-state fermentation (SSF), utilizing orange peel, wheat bran, and bagasse in equal levels as inducible substrates. *A. fumigatus* is a filamentous opportunistic fungus [88,89,90,91,92] which has showed antifungal impacts against fungal phytopathogens [93,94,95,96]. Fan et al. [97] reported that both *A. flavus* TL-F3 and *A. niger* TL-F2 can promote ryegrass biomass as well as the growth of ryegrass roots, as well as inducing an increase in the content of cell wall and soluble Cd fractions in ryegrass shoots, which make them appropriate as a promising approach to enhancing phytoremediation of Cd-contaminated environments.

In one experiment, it has been reported that *A. pseudopiperis* SDBR-CMUI1 and *A. chiangmaiensis* SDBR-CMUI4 can solubilize Ca_3_(PO_4_)_2_ and improve the growth of onion (*Allium cepa* L.) and Arabidopsis (*Arabidopsis thaliana* (L.) Heynh) [98]. Inoculation of stem cuttings of sugarcane (*Saccharum officinarum* L.), and cassava (*Manihot esculenta* Crantz) with *A. pseudotubingensis* and *A. pseudopiperis* and supplementation with Ca_3_(PO_4_)_2_ showed significantly increased root and shoot dry biomasses, root lengths, cellular inorganic phosphate contents, and chlorophyll concentrations [99]. *A. egyptiacus* and *A. chevalieri* have a significant function in increasing plant immunity by promoting antioxidant enzymes, proline, and phenols, and reducing oxidative stress in a biocontrol approach to *Alternaria* leaf spot disease in *Vicia faba* plants [100]. *A. fischerianus* is well-known because of its status as a spoilage fungus, being prevalent in pasteurized food products, raw materials, and soil, and it can be found under a broad range of environmental conditions [101,102,103,104,105]. Prado et al. [106] reported that application of *A. flavipes* (ATCC^®^ 16814^TM^) could increase the dry mass and root length of *Eucalyptus*, and it can be considered a plant biostimulant due to its nontoxic, novel, and eco-friendly characteristics. Baron et al. [64] suggested that *A. brasiliensis* and *A. sydowii* can promote the efficiency of P absorption and increase P content in shoot dry matter by utilizing fertilization. It has been reported that *A. niger* v. Tiegh is a multifunctional fungus which is capable of potassium solubilization, phosphate solubilization [107,108,109], and phytohormone production [110] (Cihangir 2002).

In one experiment *A. sydowii* (F112) and *A. brasiliensis* (F111) improved phosphorus absorption by cotton plants [111]. *A. terreus* is widespread throughout the world, and found in compost heaps, deserts, and arable soil [112,113,114,115]. It has an important function in suppression of *Rhizoctonia solani* in both *Vicia faba* and *Phaseolus vulgaris*, and is an important alternative to synthetic chemical pesticides [116]. It has been reported that *A. terreus* induced higher total chlorophyll and improved root and shoot length in tomato plants [117], as well as being the main reason for improvement in sunflower yield and growth parameters and improving sunflower resistance to stem-rot disease. It could promote the growth of barley seedlings due to its P-solubilization capacity [118], and it could produce dihydroisocoumarins, which could be considered an important plant growth promotion factor [119]. *A. versicolor* also has an important role in promoting plant growth, and it can produce a wide range of secondary metabolites [120,121,122,123]. Sun et al. [124] observed that *A. oryzae* acts as both a plant growth promoter and an endophyte in radish (*Raphanus sativus*), as well as providing protection against *Plutella xylostella*, which is its major herbivore, by increasing its mortality and negatively influencing its fitness factors. Jiao et al. [125] concluded that *A. oryzae* Y-29 (AO Y-29) could increase the production of flavonoid aglycones including apigenin, genistein, luteolin, biochanin A, and isorhamnetin as well as promote yield and yield components of pigeon pea (*Cajanus cajan* L.).

It has been reported that the application of *A. awamori* had positive effects on the growth and seed production of chickpea plants, and its treatments increased seed number, shoot weight, and seed weight [126]. Ismail et al. [127] found that association of *A. japonicus* with sunflower and soybean could promote plant biomass and yield components under high-temperature stress, and it can be applied as a thermal stress alleviator in semi-arid and arid regions. *Aspergillus* spp. R_3_ could significantly decrease the severity of sheath blight disease of rice seedlings infected with *Rhizoctonia solani* Kuhn [128], and the glucose oxidase found in *A. tubingensis* has been reported to inhibit *Fusarium solani* growth and spore production in tomato plants due to its high antifungal activities [129], while *A. violaceofuscus* has been found to be an important candidate for improving the yield, proline content, relative water content, and catalase activity of tomato plants in saline-stressed soils [130]. Zhu et al. [131] reported that *A. flavus* NJC04 can be used to mitigate the adverse symptoms of *Sclerotinia sclerotiorum* in soybean.

## 4. *Aspergillus fumigatus* sp.

This is one of the most important species of fungus in the genus *Aspergillus*. *A. fumigatus* is a thermophilic fungus, it grows well up to about 50 °C, and its conidia survive the higher temperatures of self-heating compost. Due to the small size of the conidia, spores reach the alveoli in the lungs, and it can germinate into hyphae and cause endothelial damage. It may produce gliotoxin, which can inhibit phagocytosis; moreover, it can also infect the eye, skin, and other organs, causing aspergilloma and allergic bronchopulmonary aspergillosis. Colonies of it are velvety or powdery at first, changing to smoky green, and the reverse side is white to tan. The conidiophore is smooth, with single (uniseriate) phialides, usually covering the upper half of the vesicle parallel to the axis of the stalk. Khan et al. [132] reported that GMH-1a, which was newly identified as a new strain of *A. fumigatus* LH02 via phylogenetic analysis of the 18S rDNA sequence, revealed the presence of different gibberellins (GAs) such as GA_12_ (9.8 ng/ml), GA_9_ (1.2 ng/ml), and GA_4_ (24.8 ng/ml), and inoculation of soybean plants with GMH-1a could significantly promote shoot freshness, shoot length, dry biomass, chlorophyll contents, leaf area, and photosynthetic rate under salt stress; moreover, GMH-1a-inoculated plants have shown higher salicylic acid, proline, and jasmonic acid contents, and lower endogenous abscisic acid (ABA). GMH-1a, a new strain of *A. fumigatus* LH02, has been suggested for soybean cultivation as it can enhance isoflavone contents, increase plant growth, and improve tolerance of plants under salt stress [132]. Wu et al. [133] found that components which were identified from the coculture of *Alternaria alternata* and *A. fumigatus* associated with *Coffea arabica* have shown antifungal characteristics.

The antifungal components of *A. fumigatus* LBKURCC269 have been reported by Octarya et al. [134]. In their experiment, antifungal activity was determined by the disc diffusion method, the profiles of secondary metabolites from every single culture and coculture were analyzed by liquid chromatography–mass spectrometry (LC/MS-MS), and the highest antifungal activity test results were obtained from cocultured fermented ethyl acetate extract of *B. paramycoides* LBKURCC218 with *A. fumigatus* LBKURCC269, with a clear zone of 25.67 ± 0.59 mm [134]. Moreover, the results of LC/MS-MS analysis revealed that bioactive components which are only produced during the coculture of bacteria and fungi but do not appear in a single culture are *rac*-Etomidate, 2-Ethyl-2-phenylmalonamide, n-Hexanamide, and 7-(tert-butyl)-4-imino-1,2,3,4,5,6-hexahydropyrido[3,4–d]pyridazine-1,5-dione [134]. Mehmood et al. [135] reported that flavonoids and zuxin are among the main players in the complex chemical dialogue between plant roots and fungal endophytes, namely with *A. fumigatus*. In their trial, the strain was isolated from the leaves of *Withania somnifera* and was identified as greenish *Aspergillus fumigatus* by the 18S rDNA sequence; the strain effectively colonized maize roots and increased its growth; furthermore, the application of IAA in combination with endophytes increased root colonization by 90% in the zone of cell division, 66% in the zone of elongation and 30% in the zone of maturation [135]. It is also a major air-borne fungal pathogen which is able to cause various forms of disease in humans, of which invasive aspergillosis is the most dangerous. Multiple traits contribute to its pathogenicity, such as its ability to produce specific secondary metabolites such as gliotoxin, which can inhibit the host immune response; genetic mutants that inactivate gliotoxin biosynthesis attenuate *A. fumigatus* virulence [132,133]. Siderophore biosynthesis has been studied in *A. fumigatus*, and it is reported that this mold secretes two fusarinine-type siderophores, triacetylfusarinine C and fusarinine C, to capture environmental iron, and it also employs two ferrichrome-type siderophores, hyphal ferricrocin and conidial hydroxyferricrocin, for intracellular handling of iron, such as transport of iron through conidiophores for conidiation and the conidial iron stage [132,133,134,135].

## 5. *Aspergillus aculeatus* sp.

It has been reported that *A. aculeatus* has an important function as a plant growth-promoting fungus which can stimulate plant growth as well as improving the tolerance of plants to abiotic stress [136,137,138,139]. The fungus belongs to the genus *Aspergillus* [136,137,138,139,140,141,142,143]. It grows between about 10 and 42 °C, with an optimum near 30 °C, and it has high pectinolytic enzyme activity. The species produces secalonic acid D and some other minor compounds; secalonic acid D has significant animal toxicity, but a role in animal and human disease. Colonies are dark brown to black, and its heads bear phialides only. It is distinguished from *A. japonicus*, which also produces phialides only, by its larger vesicles and ellipsoidal conidia. The conidia are ellipsoidal or subspheroidal, 4–5 µm long, spinose, and borne in radiate heads. Zhang et al. [144] found that *A. aculeatus* GC-09 can be considered one of the most important options for the biocontrol of citrus postharvest blue mold caused by the pathogenic fungus *Penicillium italicum*, and its antifungal properties are due to the reduction in antioxidant enzyme activity, the accumulation of intracellular reactive oxygen species (ROS), and the disruption of cell membrane integrity. Xie et al. [145] reported that *A. aculeatus* inoculation could significantly increase heat and drought stress tolerance in tall fescue, as it can considerably influence leaf relative water content and turf quality; moreover, it can mitigate the negative effects of reactive oxygen species-induced cell damage and bursting.

Inoculation with *A. aculeatus* can improve photosynthetic efficiency under salt, drought, and heat stress conditions, and increase sugars such as talose, sorbose, fructose, and glucose, and amino acids such as glycine and proline [145]. It has been reported that *A. aculeatus* strains had notable ability to produce siderophores, indole acetic acid, and 1-aminocyclopropane-1-carboxylate deaminase, solubilize phosphate, and reduce MDA in rice roots as well as inducing higher antioxidative enzyme activities and increasing the levels of the ROS-scavenging metabolite GSH, which can increase the potential of rice plants to resist Cd stress [146]. *A. aculeatus* can be easily cultured in axenic cultures, which enables easy propagation because it has no host specificity, and it has an important impact on attenuating salt stress by producing indole-3-acetic acid (IAA) and siderophores, thus conferring stress tolerance to plants [146]. In another experiment, it has been proved that inoculation with *A. aculeatus* decreased mannose components, but enhanced both citric acid and trehalose content, which has shown its importance in the accumulation of various metabolites linked to Cd tolerance in bermudagrass (*Cynodon dactylon* (L.) Pers.) [146,147]. It has been found that *A. aculeatus* can be considered a potential biofertilizer for perennial ryegrass in sustainable agriculture, and it can solubilize K in its bound form, and increase soluble K together with enabling higher growth rate and quality [148]. *A. aculeatus* can solubilize natural forms of phosphorus, and accelerate plant uptake and utilization of phosphorus [146]. Li et al. [149] indicated that *A. aculeatus* has a special capability to counteract the negative impacts of abiotic stresses and promote plant growth in perennial ryegrass (*Lolium perenne*), as *A. aculeatus*-inoculated leaves under heat and drought stress showed lower salicylic acid and higher abscisic acid contents in comparison with non-inoculated regimes; moreover, the antioxidant activities decreased and the photosynthetic performance increased in inoculated plants under both heat and drought stress.

Under heat stress, *A. aculeatus*-inoculated plants showed higher expression of *DREB1A*, *sHSP17*.*8*, and *HSP26*.*7a* and lower expression levels of *FeSOD* and *POD47* than non-inoculated plants [150]. The capacity to dissolve insoluble phosphorus in nature of *A. aculeatus*, therefore promoting its transport, uptake and utilization, has also been reported [151]. The Cd- and salt-induced inhibition of plant metabolic activity seemed to be reduced by *A. aculeatus*, and it could increase the photosynthesis and growth rate of plants with IAA and siderophores [152,153,154,155,156]. Root colonization and association of fungal hyphae with roots resulted in promotion of plant growth, higher yields, and a reduction in Cd translocation to plant shoots under unfavorable conditions, and A. aculeatus can be easily cultivated on synthetic media, allowing for large-scale propagation in contrast to arbuscular mycorrhizal fungi [152,153,154,155,156]. Li et al. [157] found that *A. aculeatus* could positively and significantly promote the forage quality of bermudagrass and mitigate the adverse impacts of K or P deficiency stress. In their experiment, exogenous A. aculeatus significantly decreased H_2_O_2_ level activities [157].

## 6. *Aspergillus niger* sp.

This is a major filamentous ascomycete fungus, belonging to the genus *Aspergillus* [158,159], which is extensively used in food processing and the fermentation industry to produce different primary and secondary components and metabolites, such as bioactive components, organic acids, and enzymes [160,161,162,163]. *A. niger* is known as a robust microbial cell factory for organic acid production [164]. It is widely distributed in soil, decaying vegetation, food products, and indoor environments. It is one of the most industrially important fungi because of its role in the production of enzymes, citric acid, and biotechnology products. It includes species with uniseriate or biseriate conidial heads, spherical to pyriform vesicles, smooth-walled stipes and black- or near-black-colored conidia. Its conidial heads are large, globose, and dark brown, becoming radiate and tending to split into several loose columns with age. Conidiophore stipes are smooth-walled and hyaline or turning dark towards the vesicle. The conidial heads are biseriate with the phialides borne on brown, usually septate metulae. The conidia are globose to sub-globose, dark brown to black and rough-walled. Both *A. niger* and *A. ochraceus* can produce carcinogenic ochratoxin A (OTA), which is a polyketide derivative formed by connecting a dihydrocoumarin moiety and L-phenylalanine by an amide bond [158,159,160].

It is also known as black mold and causes infection in vegetables and food. In immunocompromised patients, it can cause invasive pulmonary aspergillosis, and it may also cause otomycosis. Colonies of it are woolly at first and white to yellow, turning dark brown to black. The reverse side is white to yellow, and the conidiophore is of variable length; the phialides are biseriate and arranged in two rows covering the entire vesicle, shaping a radiate head. Many studies have reported the positive influence of *A. niger* as a fungal microbial fertilizer to improve the vegetative growth of sorghum after inoculation through means of bio-carriers [165,166,167]. Tian et al. [168] reported that cell-free *A. niger* fermentation broth (AFB) could mitigate oxidative stress in maize seedlings, alleviate the oxidative damage caused by low-P stress, and improve the resistance to low-P stress in maize seedlings. Mundim et al. [130] reported that *A. niger* could significantly increase the production of shoot fresh mass of kale, lettuce, watermelon, scarlet eggplant, tomato, melon, and pepper. It could promote the growth of both maize and coffee seedlings [169]. *A. niger* GH1 could increase the antioxidant activity and polyphenolic content of the castilla rose (*Purshia plicata*), which is a native plant of the semi-desert region in northeast Mexico [170]. Different strains of *A. niger* have been discovered to be endophytic, increasing plant growth by modulating plant secondary metabolites or plant hormones [171,172,173,174]. Staib et al. [175] reported that *A. niger* and *A. fumigatus* have positive effects on epidemiological and biological aspects of both clivia (*Clivia miniata*) and cactus (*Epiphyllum truncatum*). They have also reported that the epidemiological significance of these findings is of interest with a view to the present efforts to control aspergillosis in the environments of susceptible persons, and observations of preferential growth of certain Aspergillus species in the soil of defined plants under defined conditions raise problems relating to soil microbiology. The impacts of *A. niger* on the yield and yield components of various plants are shown in Table 1.

## 7. *Aspergillus terreus* sp.

This is also known as *A. terrestris*, which is a fungus found worldwide in soil. Akhtar et al. [169], in an experiment which was conducted in an open field, found that a triple treatment of *A. terreus* inoculation with arbuscular mycorrhizal and soil amendment with compost could increase fruit setting, biomass production, carbohydrate content, and biomass production of tomato plants, but reduced root membrane permeability, peroxidase, catalase, and proline, and it can be applied for reclamation of Cr-polluted soils; moreover, in their experiment, dual inoculation with a mixed culture of arbuscular mycorrhizal fungi and *A. terreus* aided nutrient uptake, particularly of P in tomato, and increased photosynthetic efficiency by quantitatively increasing photosynthetic pigment production.

*A. terreus* occurs commonly in soil and is commonly reported as a pathogen of animals and humans. Its conidial heads are compact, columnar and biseriate. The metulae are as long as the phialides. Conidiophore stipes are hyaline and smooth-walled. The conidia are globose to ellipsoidal, hyaline to slightly yellow and smooth-walled. As a fungal pathogen of animals, humans and plants, it can cause foliar blight disease, and it also has intrinsic AmB resistance in both in vitro and in vivo conditions. This intrinsic resistance mechanism has presented a challenge to antifungal therapy for the treatment of fungal infections. Morphologically, it can be distinguished from other *Aspergillus* spp. by its aleuroconidia, which are produced in both in vivo and in vitro conditions, and which also have anticancer bioactive components such as polyketides. Colonies of it are usually velvety cinnamon brown, the reverse side is white to brown, the conidiophores are smooth and short, the phialides are in two rows and compactly columnar, and the conidia are very small. In one experiment, inoculation of pearl millet seedlings with *A. terreus* under NaCl stress significantly increased flavonoid, phenol, soluble sugar, chlorophyll, and relative water contents and IAA production [195,196]. The endophyte *A. terreus* has a positive influence on plant growth parameters, with positive effects on IAA, hydrogen cyanide, flavonoid and phenol production, which showed that it could be considered a helpful inoculant to increase yield and productivity in a sustainable farming system. Choi and Ahsan [165] reported that *A. terreus* ANU-301 successfully inhibited the growth of various plant pathogenic fungi: *Alternaria alternata*, *Colletotrichum acutatum*, and *Fusarium oxysporum*.

It has also been reported that *A. terreus* can be used as an alternative to synthetic chemical pesticides that can negatively influence human and environmental health, as it is an effectual factor in controlling the suppression of *Rhizoctonia solani* in legumes, particularly *Vicia faba* and *Phaseolus vulgaris*, it can reduce oxidative damage such as by reducing hydrogen peroxide and malondialdehyde levels, and it can improve antioxidant defense systems such as catalase, peroxidase, polyphenol oxidase and superoxide dismutase enzyme activities, as well as improve photosynthetic pigments [197,198]. *A. terreus* effectively and positively inhibits the phytopathogenic fungi *Pythium ultimum*, *Rhizoctonia solani*, and *Botrytis cinerea* [199,200,201]. It also has beneficial effects on disease control in soil infested with *P. deliense*, which may cause damping-off disease in maize [202]. Yoo et al. [197] concluded that *A. terreus* JF27 has unique potency as a growth promoter and could be applied to control bacterial speck disease by promoting resistance in tomato plants; induced resistance is triggered via jasmonic acid/ethylene-dependent or salicylic acid-dependent pathways, and is categorized into two major groups: induced systemic resistance (ISR) and systemic acquired resistance (SAR) [203,204,205]. Waqas et al. [206] showed that the endophytic fungi *A. terreus* and *Penicillium citrinum* promote growth and mitigate stem rot in sunflowers.

It has been reported that *A. terreus* was isolated from the rhizosphere of soybean crops, and it can be applied as a bioagent for suppression of charcoal rot in soybean [207,208]. Khan et al. [132] reported that an endophytic fungus, *Aspergillus terreus* BTK-1, from the root of *Chenopodium album* inoculated with wheat seedlings showed higher concentrations of gibberellins and IAA, whereas it showed low contents of abscisic acid, and the inoculated seedlings also showed high activity of reduced glutathione and low activity of peroxidase, catalase, and ascorbate under salt stress. Application of *A. terreus* on either infected or healthy eggplants revealed various responses in terms of density and the number of polyphenol oxidase (PPO) and peroxidase isozymes (POD) of eggplant (*Solanum melongena*) seedlings, and it can improve protein, photosynthetic pigments, carbohydrates and enzymatic activity, which makes it a suitable alternative in the treatment of fungal diseases, especially early blight [209,210].

## 8. *Aspergillus flavus* sp.

*A. flavus* is a common filamentous fungus extensively found in air, soil, and crops [211,212] which belongs to the family of Aspergillaceae in the phylum of Ascomycota [213,214]. It has a worldwide distribution and normally occurs as a saprophyte in soil and on many kinds of decaying organic matter; however, it is also a recognized pathogen of animals and humans. Its conidial heads are typically radiate, later splitting to form loose columns which are biseriate but have some heads with phialides borne directly on the vesicle. Its conidiophore stipes are hyaline and coarsely roughed, which is usually more noticeable near the vesicle. The conidia are globose to sub-globose, pale green and conspicuously echinulate, and some strains produce brownish sclerotia. It can develop on different crops such as spices, dried fruit, oilseed, and maize, and it is also the main producer of aflatoxin B_1_ (AFB_1_) [215,216].

Its reproductive cycle includes a sexual developmental phase and an asexual growth phase [217,218]. It is a yellow-green saprophytic aerobic fungus mainly found in the form of mycelium and conidia [219,220,221,222]. *A. flavus* is also one the major producers of aflatoxin and can contaminate a wide range of agricultural commodities, either in storage or in the field [211]. It is also reported to be associated with many diseases in humans, the most severe of which is invasive aspergillosis [211,212]. It can also cause diseases in crops as well as insects [213,214]. It is reported that once it has contaminated food, it produces large amounts of conidia, and owing to their small volume, the conidia can become suspended in the air and spread easily among foods and grains in storage facilities [213,214]. It can also produce vast quantities of aflatoxins during infection, which contaminate the grains, leading to great economic losses and health damage to animals and humans [211,212,213,214]. A. flavus can also produce a wide array of secondary metabolites in addition to aflatoxins, and these secondary components are being investigated for possible pharmaceutical applications [211,212,213,214]. As a thermotolerant fungus that can grow even at 48 °C and which can cause infection in cereals, legumes and grains, it can cause sinusitis, keratitis, otitis, and systemic and pulmonary infections in immunocompromised patients as well as being the main cause of cutaneous infection and non-invasive fungal pneumonia. Colonies of it are velvety and yellow to green or brown, and the reverse side is golden to red-brown. The conidiophores are variably rough, long, pitted, and spiny; the phialides are uniseriate and biseriate, cover the entire vesicle, and point out in all directions [214,215,216,217,218,219,220,221,222].

The inoculation of tomato with *A. flavus* can also enhance total protein, phenolic, carbohydrate, flavonoid, and chlorophyll contents as well as other secondary metabolite contents in treated plants [223,224]. It has been proved that A. flavus can solubilize hydroxyapatite, tri-calcium phosphate (TCP), and di-calcium phosphate (DCP) [225,226,227,228]. *A. flavus* exhibited notably high IAA [229,230,231,232] and gibberellin production [233,234]. Rawat et al. noted the high potency of *A. flavus* in solubilization of phosphates [235,236]. Under solid-state fermentation using *A. flavus*, jambolan (*Syzygium cumini* L) [237,238,239,240] fruit pulp can be used as an appropriate substrate for the production of antioxidant components [241,242,243,244,245,246,247]. Zhu et al. [236] found that non-aflatoxigenic NJC04 was shown to contain important deletions in the aflatoxin and cyclopiazonic acid biosynthesis pathways, and this strain inhibited mycelial growth and sclerotia formation of soybean pathogen *Sclerotinia sclerotiorum*. It also showed various antifungal mechanisms against *S. sclerotiorum*, including competition for space and nutrients, and the production of antifungal kojic acid, which was detected at 87 ± 4 mg/L in the culture medium, and NJC04 colonized soybean plants during long periods and did not cause necrotic lesions [237].

## 9. Conclusions and Future Prospects

Interactions between microorganisms and plants are prevalent and important, as they play a considerable part in the functioning of agricultural systems. Endophytic fungi are very important in agricultural sciences because of their synergistic association with plants and their high ability to stimulate growth because of their synthesis of phytohormones that respond to biotic and abiotic stressors, which make them unique options as biofertilizers and biostimulants as well as biocontrol factors against different types of diseases. Endophytic fungi can also promote plant yield and other characteristics by boosting nutrient availability, and promote their lateral formation. Different types of plant growth-promoting fungi can lessen the importance of and the need for fertilizers that have been proven to have highly adverse effects on the environment due to the fungi’s abilities to promote yield and plant development both directly and via the synthesis of various phytohormones such as ethylene, cytokinins, gibberellins, and auxins, as well as acquisition of nutrients like P, Ca, K, and Na or induction of systemic resistance to abiotic and biotic environmental stresses by production of secondary metabolites and bioactive components. The genus *Aspergillus* is applied in different forms and treatments for its beneficial impacts on plants, such as in plant growth, promotion, and development. It can enhance growth promotion through the production of biologically active components, including gibberellins, auxins, and various phytohormone ingredients, as well as numerous secondary metabolites. Different types of *Aspergillus* can induce beneficial effects on plant development and growth, promote systemic resistance, and alleviate the negative effects of stresses. The selection of suitable fungal strains for plant growth promotion is very important, because some strains of the genus *Aspergillus* are pathogens of some animals and crops. Some strains of *Aspergillus* can produce phytohormones such as gibberellins, auxins, and other secondary metabolites, which can promote plant growth, while some others can cause systemic resistance and notably decrease the stress experienced by plants. The most important *Aspergillus* species which have significant roles in plant growth promotion are *A. japonicus*, *A. awamori*, *A. niger*, and *A. terreus*. In conclusion, the application of plant growth-promoting fungi, especially *Aspergillus* spp., is a promising method and an environmentally friendly practice to improve the growth and final yield of plants, and novel practices using such biostimulants should be integrated into new farming systems. In future studies, more focus should be placed on the fungi’s multifunctional capabilities of phosphate solubilization and phytohormone production, which can be part of an alternative method for the development of sustainable and eco-friendly agriculture. *Aspergillus* spp. strains that do not produce aflatoxins can be considered fungal biocontrol agents in the prevention of contamination with biotoxins.

## Figures and Tables

**Figure 1:**
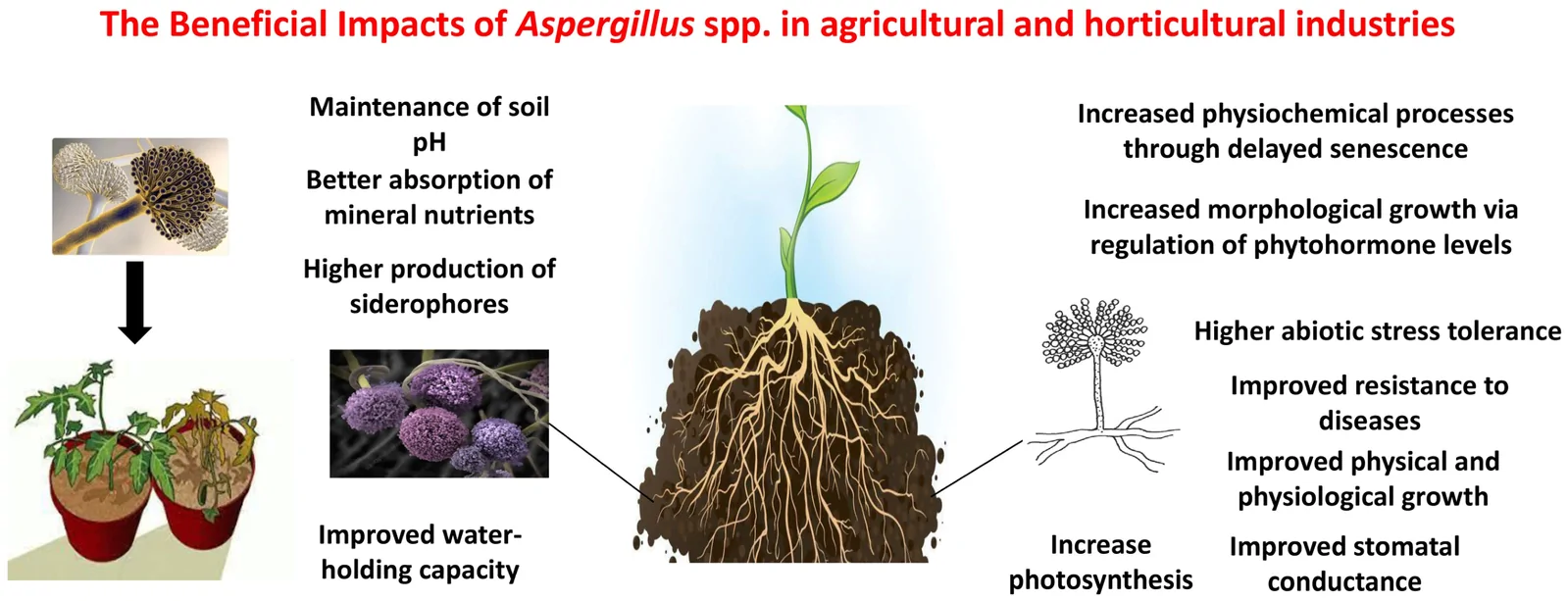
The beneficial effects of *Aspergillus* spp. in agricultural and horticultural industries.

**Table 1:** The effects of *A. niger* on the yield and yield components of various plants.

Plant	Plant Family	Key Point	References
Arrowhead plant (*Syngonium podophyllum*)	Araceae	It could increase cell wall immobilization as well as promote the subcellular compartmentalization of uranium in the root.	[176]
		Its application can alleviate the phytotoxicity of uranium entering root cells, and suppress the calcium efflux from root cells.	[176]
Carrot (*Daucus carota* L.)	Apiaceae	Its application can decrease leaf blight and soft rot.	[177]
Chickpea (*Cicer arietinum* L.)	Fabaceae	Enhanced plant growth and soil fertility as well as increased solubilization of fixed phosphates after inoculation with *A. niger* strain BHUAS01 have been reported.	[178]
Coffee (*Coffea arabica* L.)	Rubiaceae	Seed treatment with *A. niger* spores enhanced germination. It is also a biocontrol agent against collar rot (*Rhizoctonia solani*) in coffee.	[179]
		It has shown notable plant growth promotion characteristics, as it can improve the growth of coffee seedlings.	[180]
		Seedling inoculation with a granular formulation of *A. niger* induced higher stem diameter, height, root dry mass, and number of leaves.	[181]
Finger millet (*Eleusine coracana* L.)	Poaceae	Application of the *A. niger* K7 strain could positively promote the growth and yield of plants.	[182,183,184]
Groundnut (*Arachis hypogaea* L.)	Fabaceae	After inoculation, growth was suppressed in plants grown on inoculated soil, but the final yield of plants was not influenced.	[185]
Lettuce (*Lactuca sativa* L.)	Asteraceae	Lettuce plants originating from inoculated seeds revealed a higher number of leaves, plant diameter, chlorophyll content and fresh weight.	[186]
Maize (*Zea mays* L.)	Poaceae	The inoculation induced the production of a higher cumulative cob yield.	[187]
Soybean (*Glycine max* L.)	Fabaceae	Significant increases in root nodulation, growth, and final yield were reported after inoculation.	[188]
Sunflower (*Helianthus annuus* L.)	Asteraceae	Inoculation with *A. niger* improved chlorophyll content and plant biomass and height.	[189]
Tomato (*Solanum lycopersicum* L.)	Solanaceae	Its application could decrease the negative effects of Cr and Cd stress through decreasing their translocation into aerial parts.	[190]
		Its application could lead to production of higher amounts of flavonoids, proline, catalase, phenols, proteins, sugar, and ascorbic acid oxidase (AAO).	[191]
Watermelon (*Citrullus lanatus* (Thunb.) Matsum. & Nakai)	Cucurbitaceae	It can be used to suppress a seedborne disease, bacterial fruit blotch of watermelon caused by *Acidovorax citrulli*.	[192]
White clover (*Trifolium repens* L.)	Fabaceae	It could promote plant growth, and result in a higher P concentration.	[193]
Wild mint (*Mentha arvensis* L.)	Lamiaceae	It did improve menthol extraction from fresh leaves.	[194]

## Data Availability

No new data were created or analyzed in this study. Data sharing is not applicable to this article.
